# Neural oscillation in bipolar disorder: a systematic review of resting-state electroencephalography studies

**DOI:** 10.3389/fnins.2024.1424666

**Published:** 2024-08-21

**Authors:** Ziyao Su, Haoran Zhang, Yingtan Wang, Bingxu Chen, Zhizhen Zhang, Bin Wang, Jun Liu, Yuwei Shi, Xixi Zhao

**Affiliations:** ^1^National Clinical Research Center for Mental Disorders & National Center for Mental Disorders, Beijing Key Laboratory of Mental Disorders, Beijing Anding Hospital, Capital Medical University, Beijing, China; ^2^Advanced Innovation Center for Human Brain Protection, Capital Medical University, Beijing, China; ^3^The second Affiliated Hospital of Xinjiang Medical University, Urumqi, China; ^4^Faculty of Information Technology, Beijing University of Technology, Beijing, China; ^5^School of Mathematical Sciences, East China Normal University, Shanghai, China

**Keywords:** Bipolar disorder, biological markers, neural oscillation, rsEEG, spectral power

## Abstract

Bipolar disorder (BD) is a severe psychiatric disease with high rates of misdiagnosis and underdiagnosis, resulting in a significant disease burden on both individuals and society. Abnormal neural oscillations have garnered significant attention as potential neurobiological markers of BD. However, untangling the mechanisms that subserve these baseline alternations requires measurement of their electrophysiological underpinnings. This systematic review investigates consistent abnormal resting-state EEG power of BD and conducted an initial exploration into how methodological approaches might impact the study outcomes. This review was conducted in Pubmed-Medline and Web-of-Science in March 2024 to summarize the oscillation changes in resting-state EEG (rsEEG) of BD. We focusing on rsEEG to report spectral power in different frequency bands. We identified 10 studies, in which neural oscillations was compared with healthy individuals (HCs). We found that BD patients had abnormal oscillations in delta, theta, beta, and gamma bands, predominantly characterized by increased power, indicating potential widespread neural dysfunction, involving multiple neural networks and cognitive processes. However, the outcomes regarding alpha oscillation in BD were more heterogeneous, which is thought to be potentially influenced by the disease severity and the diversity of samples. Furthermore, we conducted an initial exploration into how demographic and methodological elements might impact the study outcomes, underlining the importance of implementing standardized data collection methods. Key aspects we took into account included gender, age, medication usage, medical history, the method of frequency band segmentation, and situation of eye open/eye close during the recordings. Therefore, in the face of abnormal multiple oscillations in BD, we need to adopt a comprehensive research approach, consider the multidimensional attributes of the disease and the heterogeneity of samples, and pay attention to the standardized experimental design to improve the reliability and reproducibility of the research results.

## 1 Introduction

Bipolar disorder (BD) is a prevalent clinical mood disorder affecting approximately 1% of the global population (McIntyre et al., 2020), around 0.6% of the population in China (Huang et al., 2019). BD is characterized by early onset, frequent recurrence, high morbidity and mortality, with suicide risk being particularly prominent, posing a significant challenge to public health (Nierenberg et al., 2023; Dev et al., 2024). Due to atypical symptoms during the early stages of BD and the variability of symptoms, misdiagnosis and underdiagnosis are common, delaying effective treatment increasing the burden on patients and society (Craddock and Sklar, 2013; Bauer et al., 2018). Therefore, the quest for specific biomarkers to enhance the diagnostic accuracy of BD has become an urgent need in current research, as this will aid in early identification, timely intervention, and improved patient outcomes.

Neural oscillations have gained extensive attention as a potential biological marker of BD (Howells et al., 2018; Lu et al., 2022; Bokhan et al., 2023). Oscillations are defined as the repetitive and rhythmic electrical activity that occurs spontaneously or in response to stimuli within the central nervous system (Cole and Voytek, 2017; Han et al., 2021, 2023b; Wang et al., 2023). They play key roles in brain information processing, memory formation and emotion regulation (Fitzgerald and Watson, 2018; Cao et al., 2022; Han et al., 2022; Han et al., 2023a). Previous studies have found that bipolar disorder patients exhibit disturbances in neural oscillations, suggesting a dysfunction in brain network operations (Narayanan et al., 2015; Lu et al., 2022; Andrews et al., 2023). This dysfunction is not only manifested by altered oscillation intensity within specific frequency ranges but also by disrupted synchrony of oscillations between different brain regions. On a microscopic scale, the fine balance between excitatory and inhibitory neurons is considered one of the key pathophysiological mechanisms in BD (Schaul, 1998; Sohal and Rubenstein, 2019). When an E/I imbalance occurs, neuronal spiking becomes aberrant, leading to abnormalities in the brain’s neural oscillation patterns.

**FIGURE 1:**
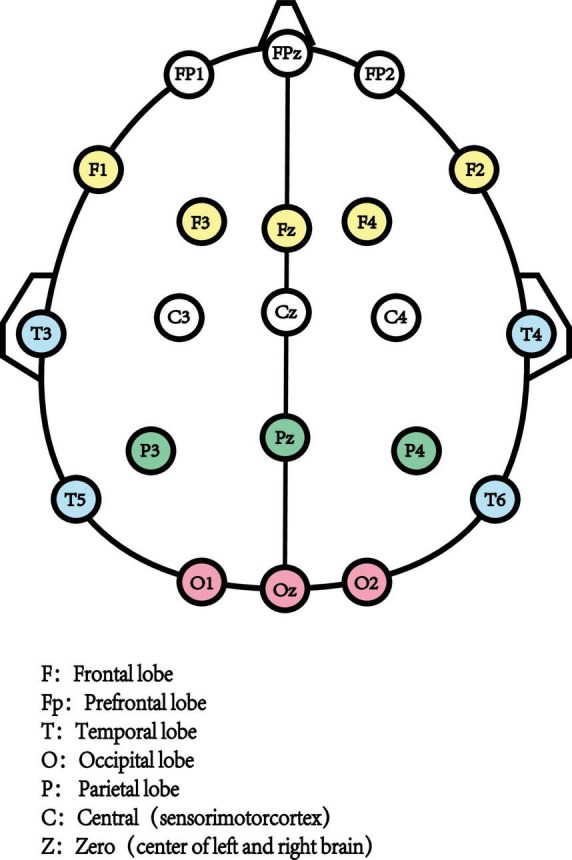
10–20 system EEG.

**FIGURE 2:**
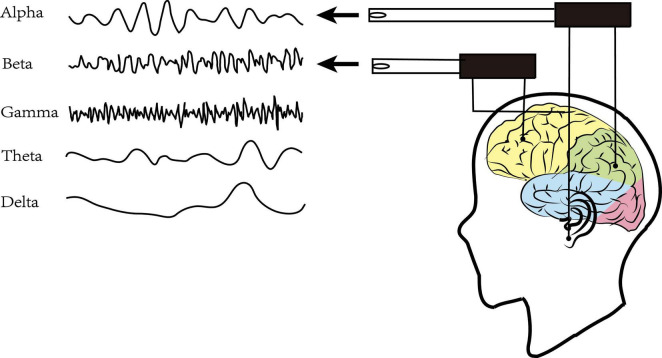
Frequency bands of brain oscillation.

Resting-state Electroencephalography (rsEEG) as a direct, non-invasive, and relatively inexpensive assessments measured the electrical field obtained from the summations at scalp electrodes of the oscillatory component generated by postsynaptic potentials in pyramidal cortical neurons (Biasiucci et al., 2019; Figure 1). The most common way to characterize rsEEG is by breaking down oscillatory signals into the spectral power of a frequency band (Figure 2). Power changes in specific frequency bands may be an indicator for an increased firing rate within certain cell populations reflecting different stages of cognitive arousal, cognitive processing, and psychopathology (Makeig et al., 2004).

Although there has been extensive recent research on the use of frequency bands in individuals with BD to investigate and understand changes in brain activity, there is no systematic review integrated the finding of BD and rsEEG. This is probably due to the heterogeneity of demographic and methodological elements. Therefore, the main purpose of our study was to find consistent abnormal resting-state oscillation patterns of BD thus providing a reliable foundation for improving early diagnosis and treatment strategies. Furthermore, we conducted an initial exploration into how methodological approaches might impact the study outcomes, underlining the importance of implementing standardized data collection methods.

## 2 Materials and methods

### 2.1 Search strategy and information source

We conducted a comprehensive search of English-language literature until March 2024 in PubMed and Web of Science, there was no limitation on publication date in search strategy. Following the Preferred Reported Items for Systematic Reviews and Meta-Analyses (PRISMA) 2021 guidelines (Page et al., 2021). We focused on samples using terms “bipolar disorder”. In terms of techniques, our interest encompassed “resting-state EEG,” “quantitative EEG,” “brain rhythms,” and “brain oscillations.” We used the Boolean expression “AND” to join the two terminologies. Moreover, the references of the selected articles were also examined to retrieve documents missed by the literature search.

### 2.2 Inclusion and exclusion criteria

The objectives of this paper and the inclusion criteria were structured based on the elements of the PICOS model (Population of interest, Interventions, Comparators, Outcomes, and Study design).

An article was included if rsEEG was assessed in a group of patients with BD. Only studies that investigated control group of health controls (HCs), either exclusively or in conjunction with other disease, were included in our review. The comparison of different states of BD was excluded. Studies employing alternative techniques, such as positron emission tomography (PET), magnetic resonance imaging (MRI), or magnetoencephalography (MEG), instead of EEG were excluded. We Use rsEEG to report spectral power in different frequency bands, studies exclusively focusing on other EEG metrics (e.g., asymmetry, coherence, functional connectivity, microstates, entropy etc.) were excluded. We only included empirical studies which written in English (Table 1).

**TABLE 1:** Inclusion and exclusion criteria of studies.

Inclusion criteria	Exclusion criteria
At least one group with BD	Experimental studies that lacked a comparison group of HCs
Studies use EEG to learn	Studies employing PET, MRI or MEG
spectral power in different frequency bands	Studies exclusively focusing on other EEG metrics (e.g., asymmetry, coherence, functional connectivity, microstates, entropy etc.)
Empirical studies	Reviews, commentaries, or meta-analysis
Written in English	Written in other languages

### 2.3 Data extraction

Information from each included article was extracted and entered into tables, the extracted data included the following information: (1) authors and year of publication, (2) demographic characteristics (sample size, sex, age, disease and medication condition), (3) recording condition (eyes closed or eyes open), (4) measures of frequency bands and range (delta- δ, theta- θ, alpha- α, beta- β, gamma- γ), (5) spectral power type utilized, (6) main findings.

The selected articles and their data have been shown in the data extraction (Tables 3, 4). whereas the study outcomes were discussed in the results section. The analysis of the results has been generally explained in the discussion part.

**TABLE 2:** Quality Assessment of Documents (NOS Scale Case–Control Studies).

study	Selection (Maximum 4 stars)	Comparability (Maximum 2 stars)	Exposure (Maximum 3 stars)
Başar et al., 2012	***	*	***
Khaleghi et al., 2019	**	**	***
Rommel et al., 2016	**		***
Narayanan et al., 2015	**		***
Clementz et al., 1994	***	*	***
El-Badri et al., 2001	***		***
Arikan et al., 2019	**		***
Kano et al., 1992	*		***
Venables et al., 2009	***		***
Kam et al., 2013	*		***

*Represents one score.

**TABLE 3:** Demographic and clinical characteristics.

Study	Subject	Sex	Age (years ± SD)	Medication	Recording
Başar et al., 2012	18BD (15BD I;3BD II) 18HC	13F; 5M 13F; 5M	31.66 ± 5.99 29.83 ± 7.77	euthymic 4w drug-free 2w	EO&EC
Khaleghi et al., 2019	21BD (11hypomanic;10depression) 18HC	11F;10M 9F; 9M	16.1 ± 1.51 16.3 ± 1.32	New onset drug-free	EO
Rommel et al., 2016	20BD I 20HC	All female	40.3 ± 7.7 36.7 ± 4.3	euthymic Uncontrolled	EO&EC
Narayanan et al., 2015	145 BP(psychotic) 56HC	60F; 85M 22F; 34M	37.07 ± 11.28 34.29 ± 12.05	uncontrolled	EO
Clementz et al., 1994	31BD 113HC	19F;12M 50F;63M	31 ± 14.2 22.5 ± 5.4	uncontrolled	EC
El-Badri et al., 2001	29BD I 26HC	10F;19M 14F;12M	30.7 ± 6.1 27.7 ± 7.0	Euthymic uncontrolled	EO&EC
Arikan et al., 2019	75BD 11HC	42F;33M 6F;5M	34.06 ± 11.12 33.80 ± 15.25	uncontrolled	EC
Kano et al., 1992	7BD 44HC	-	45.0 ± 17.0 41.5 ± 12.7	uncontrolled	EC
Venables et al., 2009	30BD 79HC	5F;25M 37F;42M	44.5 ± 9.5 43.7 ± 15.1	uncontrolled	EO&EC
Kam et al., 2013	76BD 136HC	40F; 36M 76F; 60M	41 39	uncontrolled	EO

F: female, M: male, EO: Eyes open; EC: Eyes close.

**TABLE 4:** Main quantitative EEG results.

Study	EEG variables	Delta	Theta	Alpha	Beta	Gamma
Başar et al., 2012	aSP	NA	NA	↓ (8–13Hz)	NA	NA
Khaleghi et al., 2019	aSP	↑ (0.5–4Hz)	↑ (4–8Hz)	↑ (8–13Hz)	↑ (13–34Hz)	↑ (34–45Hz)
Rommel et al., 2016	aSP rSP	NS (0.5–3.5Hz)	↑ (3.5–7.5Hz)	NS (7.5–12.5Hz)	NS (12.5–18.5Hz)	NA
Narayanan et al., 2015	Spatial weight	↑	↑	↑	↑	NS
Clementz et al., 1994	Mean of spectral power	↑ (1–3Hz)	↑ (3.125–8Hz)	↓ (8.125–13Hz)	NS Beta1 (13.125–20Hz) Beta2 (20.125–25Hz) Beta3 (25.125–30Hz)	NA
El-Badri et al., 2001	Mean of spectral power	↑ (0.5–3.9Hz)	↑ (4–7.9Hz)	↑ (8–14Hz)	↑ (14–22Hz)	NA
Arikan et al., 2019	aSP	↑ (1—4Hz)	↑ (4–7Hz)	↑ Alpha1 (8–10Hz) Alpha2 (10–12Hz)	↑ Beta1 (12–15Hz) Beta2 (15–18Hz) Beta3 (18–25Hz) High beta (25–30Hz)	↑ Gamma1 (30–35Hz) Gamma2 (35–40Hz) High gamma (40–50Hz)
Kano et al., 1992	significance probability mapping	NA	NA	↓ Alpha1 (8–9.5Hz) Alpha2 (10–12.5HZ)	NS Beta1 (13–19.5HZ) Beta2 (20–29.5Hz)	NA
Venables et al., 2009	aSP	NS	NS	NS	NS	NA
Kam et al., 2013	aSP	NS (0.5–4Hz)	NS (4–8Hz)	↓ Alpha1 (8–10Hz) Alpha 2 (10–12Hz)	↑ Beta1 (12–20Hz) Beta2 (20–30Hz)	↑ (30–50Hz)

aSP, absolute spectral power; rSP, relative spectral power; NS, not significant change; NA, not assessed.

### 2.4 Risk of bias assessment

The risk of bias in the included studies was assessed with a modified Newcastle Ottawa Scale. The case–control studies subscale was used for assessing the risk of bias. NOS provides three domains: (1) selection, (2) comparability and (3) exposure. The highest score is 9. A score from 9 to 7 indicates high quality, from 6 to 4 moderate quality, and from 3 to 0 low quality.

## 3 Results

### 3.1 Literature search and assessment of risk of bias

**FIGURE 3:**
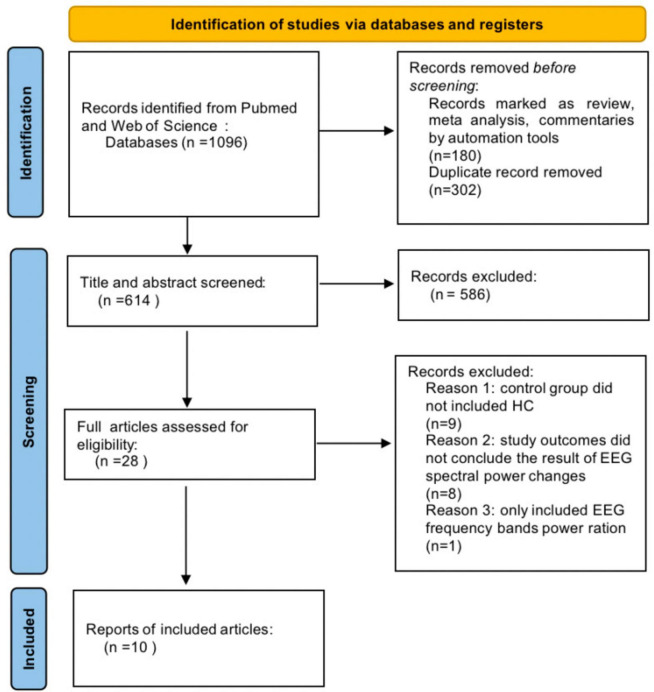
Preferred Reporting Items for Systematic Reviews and Meta-Analyses (PRISMA) flow diagram of study selection.

A total of 1096 articles were initially identified from Pub-Med and Web-of-Science databases using our search terms (Figure 3). 180 reviews, systematic reviews or meta-analyses were screened by automation tools, and 302 duplicate articles were removed. After reading the titles and abstracts, 586 articles were excluded. Upon further reading the full text, 18 articles were excluded, including 8 studies that did not address the outcomes of rsEEG spectral power changes. One study included only rsEEG frequency bands power ration (delta/alpha), instead of individual band power variations. Control group of 10 studies did not included HCs. Ultimately, 10 articles were included.

The mean score of quality assessment of the 10 studies was 5.6, indicating a moderate quality. Table 2 reports the score assigned to each article.

### 3.2 Character of the included studies

Table 3 show the demographic data and clinical characteristics of the 10 preferred studies. One articles only included women (Rommel et al., 2016), one article did not conclude sex information (Kano et al., 1992). Subjects of 2 studies were adolescents and young adults (El-Badri et al., 2001; Khaleghi et al., 2019). Two included articles control drug use (Başar et al., 2012; Khaleghi et al., 2019). In three studies, rsEEG was measured with eyes closed (Kano et al., 1992; Clementz et al., 1994; Arikan et al., 2019). However, three studies reported results for eyes open (Kam et al., 2013; Narayanan et al., 2015; Khaleghi et al., 2019), whereas four studies reported results for the eyes-closed and eyes-open conditions (El-Badri et al., 2001; Venables et al., 2009; Başar et al., 2012; Rommel et al., 2016).

Regarding frequency bands, of the 10 studies, 4 examined the conventional five frequency bands (delta, theta, alpha, beta, and gamma). Nevertheless, in 5 studies, the gamma band was excluded, 1 study only included alpha band (Başar et al., 2012). 4 studies split alpha or beta into sub-bands (e.g., alpha1/alpha2, beta1/beta2/beta3) (Kano et al., 1992; Clementz et al., 1994; Kam et al., 2013; Arikan et al., 2019). For each frequency band, power was mostly reported in terms of absolute spectral power, relative spectral power. For more details, see Table 4.

### 3.3 Reporting of frequency bands during resting state

#### 3.3.1 Resting state delta spectral power

Table 4 shows the dominant results for frequency bands power for each study.

For Delta spectral power, 4 included studies indicated increased delta power compared to HCs (Clementz et al., 1994; El-Badri et al., 2001; Narayanan et al., 2015; Khaleghi et al., 2019). Among them, Khaleghi drew the conclusion that delta frequency band of BD depression state exhibited great power in F7, F3, Fz, T3 (Khaleghi et al., 2019), and Clementz pointed out that the promote area was in C3, C4 and Cz (Clementz et al., 1994). The other 2 studies did not indicate the specific location of elevation. Three articles did not identify any statistical differences between BD and HCs patients (Venables et al., 2009; Kam et al., 2013; Rommel et al., 2016).

#### 3.3.2 Resting state theta spectral power

A comparison of HCs, six studies showed an increase in resting-state theta power (Clementz et al., 1994; El-Badri et al., 2001; Narayanan et al., 2015; Arikan et al., 2019; Khaleghi et al., 2019; Kim et al., 2020). In term of the elevated brain areas, Khaleghi pointed out the findings in F3 and T3 (Khaleghi et al., 2019) and Arikan indicated that the greatest power activities are in FP1, P3, P4, Pz, O1, F7, T3 and T4 electrodes (Arikan et al., 2019). In 2020, Kim concluded that the resting state theta power increased at the global level, with the majority of this increase occurring in the frontal lobe (Kim et al., 2020). Two studies did not report any changes in the groups in the theta frequency band (Venables et al., 2009; Kam et al., 2013).

#### 3.3.3 Resting state alpha spectral power

The outcomes regarding alpha oscillation in BD were more heterogeneous, In comparison with HCs, 4 studies showed alpha band power increased (El-Badri et al., 2001; Narayanan et al., 2015; Arikan et al., 2019; Khaleghi et al., 2019), while 4 articles indicated decreased alpha activities (Kano et al., 1992; Clementz et al., 1994; Başar et al., 2012; Kam et al., 2013). In 1992 Kano firstly proposed that alpha power was decreased in F7 (Kano et al., 1992), then Clementz suggested BD C3, C4, Cz has lesser alpha activities than HCs (Clementz et al., 1994). In 2012, Basar concluded decreased alpha power in O1, Oz, O2 (Başar et al., 2012).

#### 3.3.4 Resting state beta spectral power

In the comparison of BD to HCs, 5 studies showed an increase in beta band power (El-Badri et al., 2001; Kam et al., 2013; Narayanan et al., 2015; Arikan et al., 2019; Khaleghi et al., 2019). One study suggested that beta has greater power than HC in right temporal and left occipital particularly (El-Badri et al., 2001). Another study concluded: Beta power increased in FP1, FP2, C4, P3, O1 and F7. Beta 1 power increased in O1, and F7; Beta 2 power increased in O1 and F7; Beta 3 power increased in P3, O1 and F7 (Arikan et al., 2019).

#### 3.3.5 Resting state gamma spectral power

In comparison between BD and HCs, 3 articles indicated increased gamma power (Kam et al., 2013; Arikan et al., 2019; Khaleghi et al., 2019). One included study indicated that gamma power increased in Fp1, F8, C3, T5, T6 and O1 (Khaleghi et al., 2019), another concluded that gamma power increased in C3, F7, with gamma 1 in C3, P3, F7, gamma 2 in Cz, P3, F7 and high gamma in C3, C4, F7 (Arikan et al., 2019).

## 4 Discussion

In this systematic review, our aim was to find consistent abnormal resting-state oscillation patterns of BD. Furthermore, we try to analyze the causes and mechanisms of discrepancies based on the results, while correlating them with the summarized demographic characteristics and experimental methods. We found that BD patients had abnormal oscillations in delta, theta, beta, and gamma bands, predominantly characterized by increased power, indicating potential widespread neural dysfunction, involving multiple neural networks and cognitive processes. However, the outcomes regarding alpha oscillation in BD were more heterogeneous, which is thought to be potentially influenced by the disease severity and the diversity of samples.

### 4.1 Delta oscillation

All these studies indicated an increase in the power of the delta frequency band in the studies included in this analysis, BD was compared with HCs. Delta oscillations may reflect homeostatic and metabolic processes. The predominance of low-frequency waves in consciousness is considered a pathological state, which is often associated with cognitive decline (Uhlhaas et al., 2008).

### 4.2 Theta oscillation

All of the included studies showed an increased frequency of theta waves in BD compared with HCs, and most of them indicated that they were located in the frontal region. Theta oscillations have been shown to index cognitive performance particularly in decision-making, attention focusing and learning (Klimesch, 1999; Nokia et al., 2012). Theta oscillations depend on a balance between excitatory Glx and inhibitory GABA neurons, with alternating E/I interactions being essential for the generation and maintenance of low-frequency theta rhythms (Nigbur et al., 2011). Moreover, reduced GABA levels and increased Glx levels may exist in BD patients, thereby disrupting the rhythm balance of theta oscillations but current conclusions are not yet uniform (Chase and Phillips, 2016). Theta wave oscillations play a crucial role in synaptic plasticity regulating, spatial information processing, and memory encoding within the cortical-hippocampal circuit (Buzsáki, 2002). Abnormal discharging of hippocampal cells is a common feature among patients with psychiatric disorders, providing a reliable neurophysiological model for cognitive deficits (Olypher et al., 2006). Synaptic and neural plasticity are crucial processes for brain function and development, playing an important role in mental illness (Toro and Deakin, 2007; Narayanan et al., 2015). Neurogenesis in the hippocampus is related to synaptic plasticity, memory, and learning. Abnormal hippocampal neurogenesis is closely linked to depression and other psychiatric disorders (Kempermann et al., 2008).

### 4.3 Alpha oscillation

The results of alpha activity are different. The power of alpha band is believed to be negatively correlated with cortical activity, concentration, and thalamus metabolism (Nusslock et al., 2012). The thalamus serves as the inlet for sensory transmission and plays a crucial role in concentration. The decrease in thalamus metabolism is correlated with an enhancement in alpha power (Lindgren et al., 1999). Therefore, it can be concluded that an increase in alpha activity is associated with attention deficits and thalamic metabolic disorder.

Moreover, increased alpha activity is associated with increased depression severity. BD patients with a high depression severity are characterized by decreased neuronal excitability. This abnormal neurophysiological pattern may affect the neurodevelopmental processes in adolescents with BD. Some researchers reported that the frequency bands of adolescents undergo changes. EEG is not as same as in adults during adolescent development (Khaleghi et al., 2019). Therefore, the different age groups of participants may be a potential reason for the controversy results. Moreover, some studies noticed that drug applications have effects on oscillation dynamics (Yener et al., 2007; Başar and Güntekin, 2008). Most of the studies we included did not control drug usage. The patient’s treatment and medication may also have impact on the results which should be taken into account.

Another potential reason for the difference in the experimental results is the inconsistency of subjects included in the experiment. One study included 11 subjects with hypomanic and 10 subjects with depression (Khaleghi et al., 2019). Another study included subjects are all in an euthymic state (El-Badri et al., 2001). Khaleghi indicated BD I have greater delta power than BD II (Khaleghi et al., 2015). BD II is distinguished from BD I primarily by the absence of full-blown manic episodes. A growing body of evidence suggests that there could be neurobiological differences between BD I and BD II patients, they presumed BD I has more deficits than BD II. These factors above may be the reason for the different results. Moreover, the EEG activity varies in different emotional states (Painold et al., 2014; Abé et al., 2016). The power of the alpha band was negatively correlated with the tension-calm (TC) score, that is, the power of the alpha exhibited higher power in the calm state than in the tension state (Wyczesany et al., 2010).

Significantly, some previous works have shown that alpha rhythms could be dissected into two components in scalp EEG, which might be related to different cognitive functions (Chiang et al., 2011; Knyazeva et al., 2018). Previous results have shown that the power in these different sub-bands (upper and lower alpha) also differed in resting state (Thorpe et al., 2016). Alpha power may be considered to inversely correlate with E/I balance, which display a negative correlation with cortical activation and metabolism (Conner et al., 2011; Podvalny et al., 2015). Thus, a signature of abnormally reduced alpha power would indicate a state of increased E/I, while the ‘high-alpha’ biotype would reflect low E/I (Bresnahan and Barry, 2002; Poil et al., 2014). In healthy subjects, alpha power increases or decreases have been found to reflect cortical inhibition or excitation, respectively (Haegens et al., 2011). This demonstrates the importance of clear band allocation.

Finally, it should be noted that during resting-state, some studies only recorded EO EEG (Kam et al., 2013; Narayanan et al., 2015; Khaleghi et al., 2019), while some studies only recorded EC EEG (Kano et al., 1992; Clementz et al., 1994; Arikan et al., 2019). According to some studies, the quantitative characteristics of the alpha rhythm can only be fully understood after one takes into account the spectral power and activation intensity (inhibition of the alpha rhythm after one opens their eyes, the Berger effect). One of the informative signs of the stability of the activation reaction is a decreased alpha power in response to the opening of the eyes (the Berger effect). This is associated with information processing (Kirschfeld, 2005).

### 4.4 Beta oscillation

High beta activity is associated with cortical excitability (Rangaswamy et al., 2002) and is generally considered to be facilitating (Moore et al., 2012). Some inferences can be drawn from literature reports indicating that an elevation of emotional tension is associated with an increased in beta power, particularly in the anterior region (Jacobs et al., 1996). Alpha activity is a fundamental rhythm in the brain. The increase in occipital beta power observed during manic episodes may serve as a compensatory mechanism for dysfunctional alpha responses (Wyczesany et al., 2010). Therefore, an increase in beta-band power may be accompanied by a decrease in alpha activity.

### 4.5 Gamma oscillation

All studies reviewed herein consistently demonstrate an increase in gamma activities in individuals with BD compared to HCs. In addition, both BD and SCZ manifest overlapping symptoms, including psychotic symptoms, disorganized thinking, and depressive symptoms (Calhoun et al., 2011; Bora and Pantelis, 2016). Researches indicate that patients diagnosed with BD and SCZ exhibit notable abnormalities in neural processes involving gamma oscillations, which are both characterized by increased gamma activities (Zhou et al., 2018; Honda et al., 2020). The occurrence of gamma oscillation is contingent upon synaptic GABA neurotransmission, which is crucial for coordinating neural network activities across different brain regions (Lu et al., 2022). Cortical gamma activity plays a crucial role in processes such as sensory perception, problem-solving, and memory. Moreover, in BD the overexpression of the neuronal calcium sensor (NCS-1) protein modulates gamma band oscillation in the pedunculopontine nucleus (PPN) in a concentration-dependent manner (Urbano et al., 2014). Consequently, it can be inferred that the increased expression of NCS-1 leads to greater gamma band activity in BD patients.

Cortical gamma oscillations are believed to be generated independently of external stimuli, facilitated by GABA interneurons engaged in mutual inhibition. These interneurons generate postsynaptic potentials that oscillate at approximately 40 Hz. Consequently, gamma oscillations are considered oscillations are considered indicative of the inhibition of cortical neurons.

## 5 Critical considerations in EEG band analysis for BD research

Although the articles included in this review present promising findings, it is essential to acknowledge certain limitations identified in the analysis of frequency bands carried out in each of the reviewed investigations. Addressing these limitations is crucial for the progression of EEG research and its effective application in the study of BD.

Firstly, some researchers indicated that there are significant gender differences in EEG power between genders, with females exhibiting higher power than males (Narayanan et al., 2014). Moreover, several studies indicated that blood glutamate levels of females vary across the menstrual cycle and are negatively associated with the levels of female sex hormones (Tseng et al., 2024). It is therefore possible that males and females have different states of E/I balance. The changes may have effects on band oscillation. This suggests that inconsistencies in the sex ratio of male and female in studies may have an impact on the results. While most studies showed that the results were not due to variable gender composition across groups. Although (Rommel et al., 2016; Bokhan et al., 2023) included only female, their outcomes limit the generalizability of findings in the BD patients.

Second, different experiments have defined the age range of patients differently (e.g., 12 to 18 years considered as adolescents (Khaleghi et al., 2019), and 18 to 40 years considered as young patients (El-Badri et al., 2001). A cross-sectional study on adolescents with BD indicated that the neural defects of BD may differ between adolescents and adults (Wegbreit et al., 2014). It remains unclear whether the pathology and mechanism of BD adolescents differ from those in adults. Additionally, epidemiological research suggests that there are multiple critical periods for the onset of BD, specifically in late adolescence, around the age of 20, and between 30 to 40 years (Bellivier et al., 2003). Therefore, it is imperative for further experimental designs to consider the range of age groups. 2 experiments have defined the age range of patients differently (e.g., 12 to 18 years considered as adolescents (Khaleghi et al., 2019), and 18 to 40 years considered as young patients (El-Badri et al., 2001).

Third, although many of our preferred studies suggested there is no correlation between medication and frequency bands oscillations and did not control for patients’ drug use, some scholars indicated that the EEG results can be affected by drugs in the resting-state (Spatz and Kugler, 1982; Struve, 1987; Schulz et al., 2000; de Meneses et al., 2022). For example, lithium usually increases slow wave activities (delta, theta) and decreases alpha activities. Some antidepressants (e.g., imipramine, viloxazine) may increase beta activities and simultaneously decrease alpha activities (Saletu, 1976; Bente, 1979). Amitriptyline tends to increase delta but decrease alpha activities (Peck et al., 1979). Studies on animal models have shown that mood stabilizers can exert behavioral effects by altering synaptic E/I balance (Tseng et al., 2024). Drugs used as mood stabilizers, including lithium, valproate and lamotrigine, have significant effects on the glutamatergic system (Sanacora et al., 2008).

## 6 Conclusion

Overall, the examined studies suggested that the electrophysiological features of BD probably could play a key role in diagnosis of this disease. In studying the characteristics of neural oscillations in BD patients, we observed abnormalities in multiple frequency bands such as delta, theta, beta, and gamma, the most notable feature of which was a general increase in oscillatory power. However, the results of studies of alpha oscillation show high heterogeneity, which may be due to the different stages and manifestations of symptoms of the disease, as well as the diversity of the samples themselves. Besides, we have preliminarily explored how the findings are influenced by demographic and methodological factors. By controlling for these variables, such as age, gender, stage of disease, comorbidities, and drugs used, researchers are better able to unravel the complexity of BD and reveal potential neurophysiological markers that inform future diagnostic and therapeutic strategies.

## Data availability statement

Publicly available datasets were analyzed in this study. This data can be found here: https://pubmed.ncbi.nlm.nih.gov/?term=adult%20ADHD%20EEG&page=10.

## Author contributions

XZ: Writing–original draft, Writing–review and editing, Conceptualization, Supervision, Validation, Visualization. ZS: Writing–original draft, Writing–review and editing. HZ: Writing–original draft, Writing–review and editing. YW: Writing–original draft. BC: Writing–original draft. ZZ: Writing–original draft. BW: Writing–original draft. JL: Writing–original draft. YS: Writing–original draft, Writing–review and editing.
